# Morphine, a potential antagonist of cisplatin cytotoxicity, inhibits cisplatin-induced apoptosis and suppression of tumor growth in nasopharyngeal carcinoma xenografts

**DOI:** 10.1038/srep18706

**Published:** 2016-01-05

**Authors:** Long-Hui Cao, Hui-Ting Li, Wen-Qian Lin, Hong-Ying Tan, Lan Xie, Zhong-Jian Zhong, Jian-Hua Zhou

**Affiliations:** 1 Department of Anesthesiology, Sun Yat-sen University Cancer Center, State Key Laboratory of Oncology in Southern China, Collaborative Innovation Center for Cancer Medicine, Guangzhou, Guangdong, 510060, People’s Republic of China; 2 Department of Ultrasound, Sun Yat-sen University Cancer Center, State Key Laboratory of Oncology in Southern China, Collaborative Innovation Center for Cancer Medicine, Guangzhou, Guangdong, 510060, People’s Republic of China

## Abstract

Morphine is an opioid analgesic drug often used for pain relief in cancer patients. However, there is growing evidence that morphine may modulate tumor growth, progression and metastasis. In this study, we evaluated whether morphine modulates cisplatin-induced apoptosis in human nasopharyngeal carcinoma CNE-2 cells and whether morphine affects the antitumor activity of cisplatin on tumor growth in human nasopharyngeal carcinoma CNE-2 xenografts in nude mice. We showed that a pretreatment with morphine (1 μg/ml) inhibited the sensitivity of CNE-2 cells to cisplatin by inhibiting cisplatin-induced CNE-2 cell apoptosis, decreasing caspase-3 activity and increasing the Bcl-2/Bax ratio. However, a high dose of morphine (1000 μg/ml) had the opposite effect. We also showed that at a low dose, morphine enhances chemoresistance in an *in vivo* nasopharyngeal carcinoma (NPC) model by inhibiting cisplatin-induced apoptosis and decreasing neovascularization. Taken together, our results indicate that a low dose of morphine may lead to chemoresistance of cisplatin in NPC models *in vitro* and *in vivo* by inhibiting cisplatin-induced apoptosis and decreasing neovascularization.

Nasopharyngeal carcinoma (NPC) is a malignancy of epithelial origin with a multifactorial etiology. Although this malignant disease is rare in the Western world, it is endemic in the southern parts of China, Southeast Asia, the Mediterranean basin and Alaska^1^. Because this tumor commonly metastasizes, NPC remains the leading cause of death from head and neck cancer in South China. Some clinical trials have revealed that concurrent radiotherapy and chemotherapy improves prognoses in both early- and later-stage cases^2^,^3^. The most widely used chemotherapy regimen in the treatment of NPC is the combination of cisplatin (CDDP) and 5-fluorouracil^2^. Nasopharyngeal carcinoma often causes pain and discomfort, especially in advanced stages of the disease. Nearly half of patients with NPC present with pain at diagnosis^4^, and almost all patients experience some form of pain during radiation therapy for NPC^5^. Pain, whether arising from the cancer or its treatment, may compromise disease progress and treatment outcomes^6^. The principles of pain management should be the same as those used for other cancer-related pain, which includes the vigilant assessment of the pain and active pain therapy commensurate with cancer pain treatment guidelines. It has been clearly established that opioid analgesics improve pain control in patients with moderate and severe pain caused by irradiation to the head and neck^7^ and effectively relieve cancer pain. Morphine is a representative Opioid analgesic that is commonly used to relieve pain in cancer patients, including NPC patients. Although NPC patients often require concurrent treatment with morphine and cisplatin, little is known regarding the impact of morphine on the antitumor activity of cisplatin and its possible mechanisms.

Cisplatin forms highly reactive, charged, platinum complexes that bind to nucleophilic groups, such as GC-rich sites in DNA. This induces intrastrand and interstrand DNA cross-links as well as DNA-protein cross-links, which inhibit cell growth and result in apoptosis. Apoptosis proceeds, in part, due to the aggregation and multimerization of upstream death effector molecules that concurrently or sequentially activate the cysteinyl aspartate-specific protease (caspase) cascade^8^. Additionally, mitochondria are thought to be a major target of cisplatin, and mitochondrial DNA is heavily damaged by cisplatin^9^,^10^, leading to the loss of mitochondrial energy production, the release of a mitochondrial serine protease^11^, and subsequent cell death.

Morphine produces strong analgesic effects by stimulating opioid receptor signaling in neurons. In addition to these well-recognized effects, various studies have suggested that morphine elicits a variety of biological effects that appear to be independent of its analgesic properties and may affect cell survival or proliferation^12^. Unfortunately, the role of morphine in the regulation of tumor cell growth is not yet clear. Morphine has been demonstrated to induce the apoptosis of immunocytes^13^, cancer cells^14^, neuroblastoma cells^15^, and neuronal cells^16^. However, morphine can protect astrocytes from apoptosis triggered by apoptosis-promoting agents^17^ and promote the growth of tumor cells^18^,^19^. No studies have examined the effects of morphine on CDDP chemotherapy sensitivity in NPC. In this study, we aimed to investigate the role of morphine in NPC chemotherapy using CDDP *in vitro* and *in vivo*.

## Results

### Effect of Different Concentrations of Morphine on Cisplatin-treated CNE-2 Cell Viability

**Figure 1:**
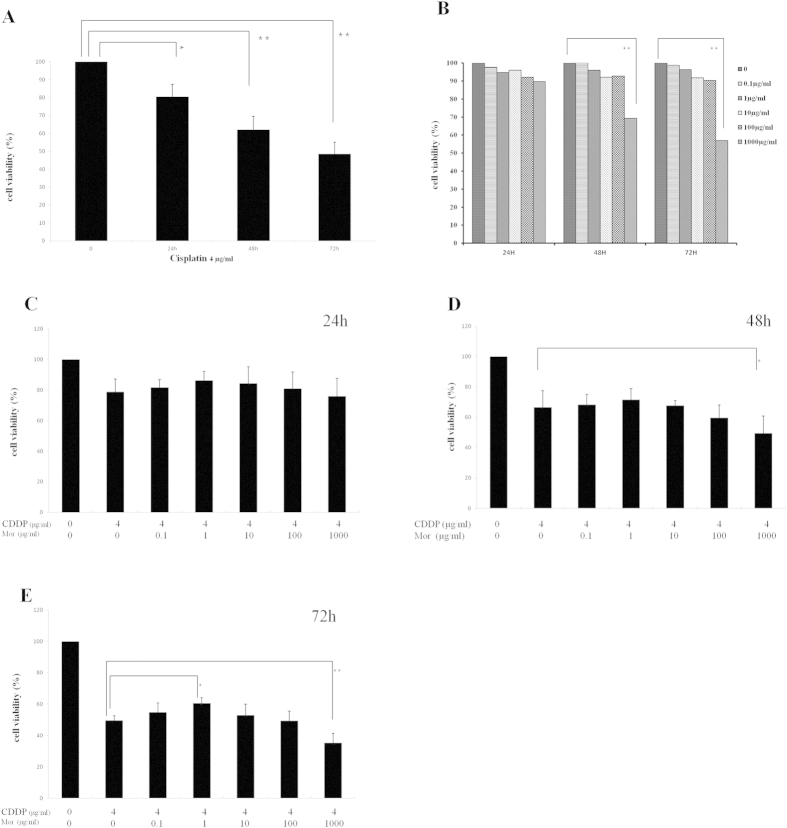
Effect of different concentrations of morphine on cisplatin-inhibited proliferation of CNE-2 cells. (**A**) Cells were treated with cisplatin (CDDP) 4 μg/ml for 24 h, 48 h, and 72 h. (**B**) Cells were treated without or with morphine (Mor) (0.1 μg/ml, 1 μg/ml, 10 μg/ml, 100 μg/ml or 1000 μg/ml) for 24 h, 48 h, and 72 h. (**C**) Cells were treated with cisplatin (CDDP) 4 μg/ml for 24 h in the presence of an increasing concentration of morphine (Mor). (**D**) Cells were treated with cisplatin (CDDP) 4 μg/ml for 24 h in the presence of an increasing concentration of morphine (Mor). (**E**) Cells were treated with cisplatin (CDDP) 4 μg/ml for 24 h in the presence of an increasing concentration of morphine (Mor). Cell viability was determined using the MTT assay as described in the Materials and Methods section. *P < 0.05, **P < 0.01. Error bars represent the mean ± SD of triplicates.

To demonstrate that cisplatin inhibits CNE-2 cell proliferation, we first treated CNE-2 cells with cisplatin (4 μg/ml) and analyzed the cell viability using an MTT assay after 24, 48 and 72 hours. Cisplatin inhibited the growth of CNE-2 cells in a time-dependent manner (Fig. 1A). Then we investigated whether morphine alone can affect the apoptosis of CNE-2 cells. After incubation with morphine (1000 μg/ml) for 48 h and 72 h, the cell viability decreased significantly. However, after incubation with different **c**oncentration of morphine including 0.1 μg/ml, 1 μg/ml, 10 μg/ml and 100 μg/ml, the cell viability did not changed significantly (Fig. 1B). At last we investigated whether morphine was able to affect the sensitivity of CNE-2 cells to cisplatin chemotherapy. The cells were pretreated with morphine for 1 hour, followed by an incubation with cisplatin for another 3 days. Our results showed that a low dose of morphine inhibited the sensitivity of CNE2 cells to cisplatin, but a high dose of morphine enhanced the sensitivity of CNE2 cells to cisplatin after 72 hours *in vitro*. Although no significant effects were observed at 48 h (Fig. 1D), treatment with morphine (1 μg/ml) significantly abolished the cisplatin-induced loss of cell viability at 72 h (Fig. 1E). However, a high dose of morphine (1000 μg/ml) significantly increased the loss of cell viability induced by cisplatin at 48 h and 72 h. At 24 h, morphine (1 μg/ml or 1000 μg/ml) did not obviously alter cell viability (Fig. 1C). Moreover, morphine (0.1 μg/ml, 10 μg/ml or 100 μg/ml) did not show any effect on the cisplatin-induced decrease in cell viability at 24 h, 48 h and 72 h (Fig. 1C–E).

### Effect of Different Concentrations of Morphine on Cisplatin-induced Apoptosis in CNE-2 Cells

**Figure 2:**
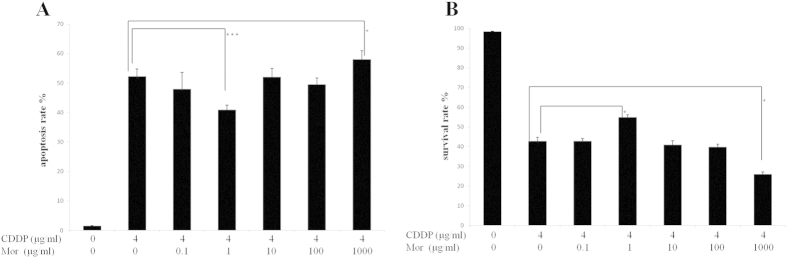
Effect of the different concentrations of morphine on cisplatin-induced apoptosis in the CNE-2 cells. Cells were treated with cisplatin (CDDP) 4 μg/ml for 72 h in the absence or presence of an increasing concentration of morphine (Mor). Thereafter, the cells were stained with Annexin V/PI and detected by fluorescence microscopy. (**A**) The apoptosis rate of the cells was determined as described in the Materials and Methods section. (**B**) The survival rate of the cells was determined as described in the Materials and Methods section. *P < 0.05, ***P < 0.001. Error bars represent the mean ± SD of triplicates.

It is generally accepted that cancer cells treated with cisplatin have reduced cell viability as a result of increased apoptosis. Thus, we examined whether the different concentrations of morphine or its absence for 72 h affected apoptosis in cisplatin-treated CNE-2 cells. The percentage of cells positive for annexin V/PI was significantly decreased following treatment with morphine (1 μg/ml) (Fig. 2A), indicating that the apoptosis rate decreased after the morphine pretreatment (1 μg/ml). Moreover, when we focused on survival rate, we found that cisplatin-induced CNE-2 cell apoptosis was apparently recovered by morphine (1 μg/ml). However, the cell survival rate decreased and the apoptosis rate increased following treatment with a high dose of morphine (1000 μg/ml) (Fig. 2B). Compared with cisplatin alone, there were no significant differences in either the apoptosis or survival rates in the CNE-2 cells following treatment with morphine (0.1 μg/ml, 10 μg/ml or 100 μg/ml).

### Expression of Bcl-2, Bax and the Activation of caspase

**Figure 3:**
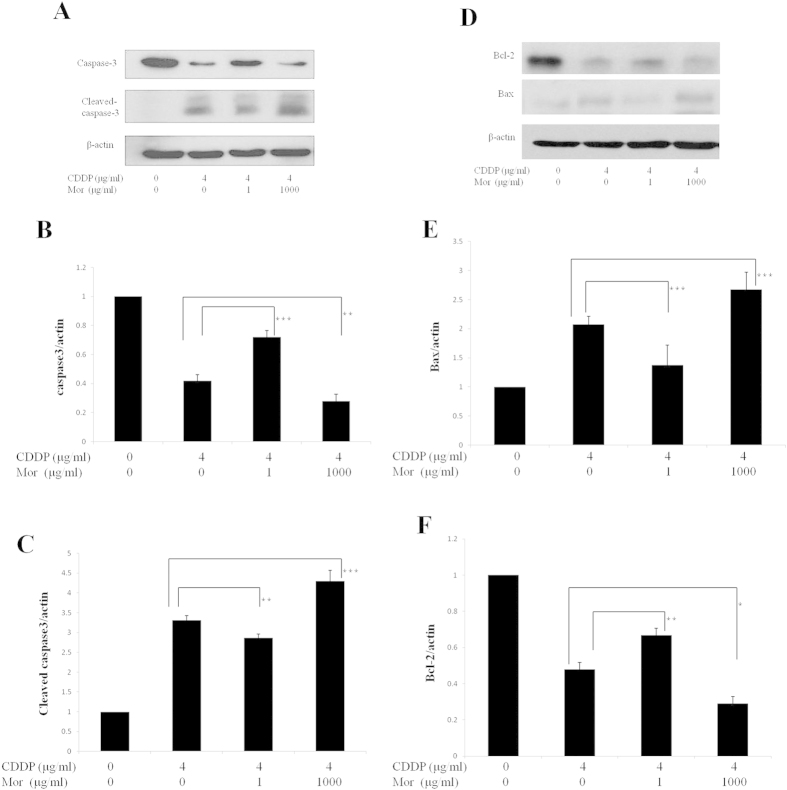
Effect of different concentrations of morphine on cisplatin-induced caspase-3 activation and the changes in the Bax and Bcl-2 protein levels in the CNE-2 cells. Cells were treated with either 4 μg/ml cisplatin (CDDP) alone or with 4 μg/ml cisplatin (CDDP) in combination with 1 μg/ml morphine (Mor) or 1000 μg/ml morphine (Mor) for 72 h. Extracts from cells were subjected to SDS/PAGE (12% gels) and immunoblotted with (**A**) anti-procaspase-3 or anti-(cleaved caspase-3). (**B,C**) The percent of relative intensity obtained from the corresponding western blots. (**D**) Anti-Bax or anti-Bcl-2 antibodies. (**E,F**) Percentage of relative intensity obtained from the corresponding western blots. Anti-β-actin antibodies were used as a control for equal loading. *P < 0.05, **P < 0.01, ***P < 0.001. Error bars represent the mean ± SD of triplicates.

Members of the Bcl-2 family of proteins control and regulate the apoptotic mitochondrial events^20^. To further evaluate the mechanism by which morphine affects cisplatin-induced CNE-2 cell apoptosis, we examined the changes in protein expression of apoptosis-related genes, including Bcl-2 and Bax. After 72 h of treatment with cisplatin (4 μg/ml), a clear reduction of Bcl-2 was observed and Bax levels were significantly increased (Fig. 3D–F). Compared to cisplatin alone, the combined treatment of morphine (1 μg/ml) and cisplatin (4 μg/ml) increased the expression of Bcl-2 but decreased the expression of Bax. In contrast, combining morphine (1000 μg/ml) with cisplatin (4 μg/ml) had the opposite effect, as the levels of Bcl-2 decreased and the levels of Bax increased (Fig. 3D–F).

Apoptosis is largely controlled by a family of intracellular cysteine proteases known as caspases, which are activated by 2 major signaling routes: the extrinsic receptor pathway and the intrinsic mitochondrial pathway. Caspase-3 is considered to be the most important of the executioner caspases and is activated by any of the initiator caspases (caspase-8, caspase-9, or caspase-10)^21^. To evaluate the possible mechanism by which morphine interacts with caspase, we assessed the effect of treatment with cisplatin alone and in combination with morphine (1 μg/ml or 1000 μg/ml) on the activation of caspase-3 in CNE-2 cells. After the CNE-2 cells were treated with cisplatin (4 μg/ml) for 72 h, the levels of the active form of caspase-3 increased (Fig. 3A–C). In the CNE-2 cells pretreated with morphine (1 μg/ml), the active form of caspase-3 was significantly decreased compared to cells treated with cisplatin alone. The higher dose of morphine (1000 μg/ml) significantly increased the levels of the active form of caspase-3 (Fig. 3A–C).

### Effect on Xenograft Tumors

**Figure 4:**
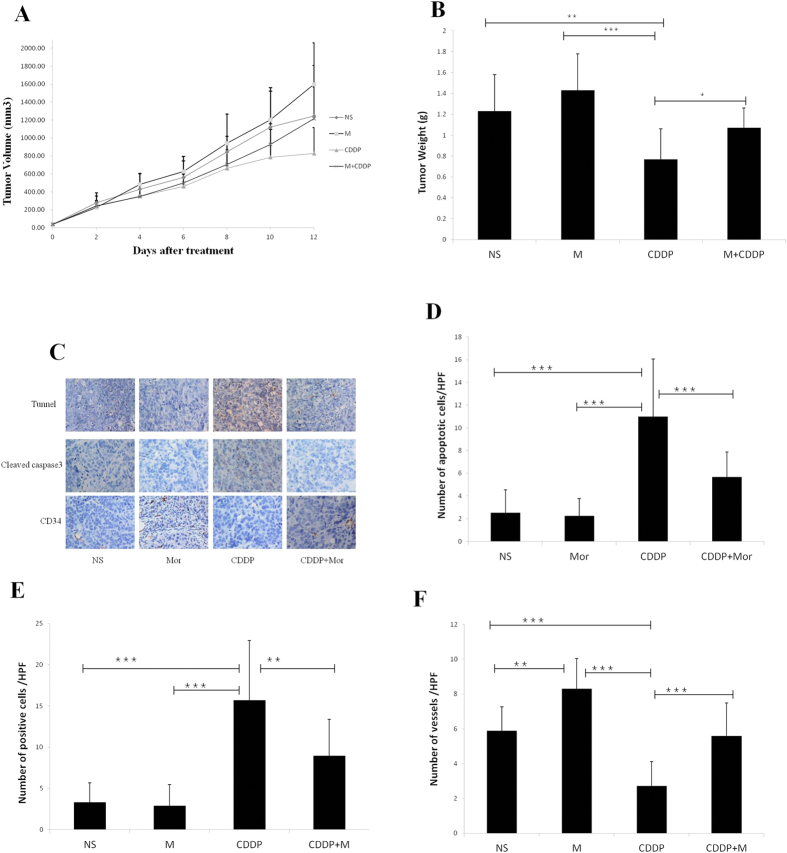
Morphine inhibited the antitumor effect of cisplatin on tumor growth of human CNE-2 xenografts in nude mice. (**A**) The effects of Mor, cisplatin (CDDP) or their combination on tumor growth in CNE-2 xenograft-bearing nude mice. The saline solution treatment served as a control. The tumor length diameter (A) and width diameter (**B**) were measured every 2 days. The tumor volumes were calculated as (A × B^2^)/2. (**B**) Comparison of the tumor weight of four groups. (**C**) TUNEL staining, cleaved-caspase-3 immunohistology and CD34 immunohistology of the CNE-2 xenograft tumor sections after the treatment. (**D**) The number of TUNEL-positive cells per × 40 high-power field (HPF) is indicated. Numbers indicate the average of 10 mice per group. (**E**) The number of cleaved-caspase-3-positive cells per × 40 high-power field (HPF) is indicated. Numbers indicate the average of 10 mice per group. (**F**) The number of CD34-positive cells per × 40 high-power field (HPF) is indicated. Numbers indicate the average of 10 mice per group. *P < 0.05, **P < 0.01, ***P < 0.001. Error bars represent the mean ± SD of 10 mice per group.

Because morphine affected cisplatin-induced apoptosis in the *in vitro* assays, we investigated the effects of morphine on an NPC xenograft in nude mice treated with cisplatin. According to Gupta’s method^18^, we chose a low dose of morphine (1 mg/kg) similar to the clinical dose used in patients. In the CNE-2 xenograft-bearing mice, the tumor volumes were the smallest in the group treated with cisplatin on day 12 (Fig. 4A). Compared with the normal saline group, the cisplatin group showed a decreased tumor weight on day 14 (*p* < 0.01) (Fig. 4B). In contrast, the morphine plus cisplatin group demonstrated an increase in tumor weight on day 14 compared with the cisplatin group (*p* < 0.05). No significant differences were found in the tumor weight between the normal saline control group and the morphine group. The results indicated that cisplatin (3 mg/kg) inhibited NPC tumor growth, which can be reversed by morphine (1 mg/kg).

To extend our observation, we further performed a TUNEL assay on tissue-sample sections of CNE-2 xenografts obtained from the 4 treatment groups to examine apoptosis. As expected, there were similar levels of programmed cell death in the tumors taken from the mice treated with normal saline or morphine (1 mg/kg) (Fig. 4C,D). However, the mice treated with cisplatin or morphine plus cisplatin had a significantly greater number of apoptotic cells in their tumors compared to the groups treated with saline or morphine alone. Compared to the cisplatin-administered group, the group treated with morphine plus cisplatin showed a decrease in tumor cell apoptosis (p < 0.001) (Fig. 4C,D). These results were similar to the expression of cleaved caspase-3, in which the combination-treated tumor showed fewer cleaved caspase-3-positive cells compared with the cisplatin treatment alone (Fig. 4C,E). These findings support our *in vitro* data, showing that morphine inhibited the cisplatin-induced apoptosis of CNE-2 cells.

Because morphine has been shown to induce angiogenesis in several *in vitro* and *in vivo* assays^18^,^19^, we examined whether morphine could stimulate angiogenesis in an *in vivo* CNE-2 cell NPC tumor xenograft model in mice. We observed a greater number of vessels in the morphine group at day 14 compared with the control group (p < 0.01) (Fig. 4C,F). The blood vessel number was reduced by approximately 50% in the cisplatin-treated group compared with the control group (Fig. 4C,F). However, the blood vessel number in the tumors from mice receiving the combination of cisplatin and morphine was significantly increased compared with the mice treated with cisplatin alone (p < 0.001) (Fig. 4C,F).

## Discussion

Most NPC patients receive morphine to relieve severe pain induced by the cancer itself, bone metastasis, radiotherapy or chemotherapy. Morphine is not only known to act as an analgesic via the μ-opioid receptor in the central nervous system but also has some direct effects on non-neural cells, such as endothelial cells, tumor cells, and mast cells^18^,^21^,^22^,^23^. However, the results obtained in the studies assessing cancer cell growth *in vitro* or *in vivo* are still controversial. Some reports have shown that morphine can inhibit the growth of various human cancer cell lines, including breast cancer, gastric cancer, lung cancer and prostate cancer^24^,^25^,^26^,^27^, but other studies have shown that morphine increases tumor cell growth *in vivo*^28^,^29^ and *in vitro*^30^. These contrasting results are likely associated with the different doses of morphine used, the route of administration, the different cell lines or tumor types studied, and/or plasma doses achieved at a steady state. Cisplatin is a chemotherapeutic agent that is widely used alone or in combination with other therapies as a treatment for a variety of tumor types. Cisplatin chemotherapy and adjuvant radiotherapy have become the standard treatment for NPC^31^. It is important to determine whether morphine interferes with cisplatin chemotherapy because it could pose a considerable problem for NPC patients.

For clinical patients, the plasma concentrations of morphine showed the wide ranges expected from the big difference in daily oral morphine dose, which is about from 2 nM ~ 3.5 μM^32^,^33^. But in animal models of addiction, the plasma concentration of morphine could be as high as 2.5 mm^34^. These wide range plasma morphine concentrations are similar with the concentrations we chose in our *in vitro* experiment. Our data demonstrated that a low dose of morphine inhibited the cisplatin-induced apoptosis of NPC tumor cells both *in vitro* and *in vivo*. In our *in vitro* study, the highest concentration of morphine (1000 μg/ml) used enhanced the sensitivity of CNE-2 cells to cisplatin, which is similar with the results of the effect of morphine alone on CNE-2 cells. It is almost impossible to obtain such high morphine plasma concentrations in patients, although this concentration may occur in people who have been addicted to morphine for a long time^35^. In contrast to the results obtained with high doses of morphine, we demonstrated that a low dose of morphine (1 μg/ml), which is similar to the plasma concentration administered orally for pain control in cancer patients^36^,^37^, inhibited the cisplatin-mediated apoptosis of CNE-2 cells. The western blot assay showed that the underlying mechanisms associated with the reduction of cisplatin-induced apoptosis are an increased Bcl2/Bax ratio and the inhibition of cisplatin-induced caspase-3 activity.

Apoptosis is a form of programmed cell death that results in the elimination of cells without releasing harmful substances into the surrounding tissues. Notably, apoptosis is deregulated in cancer cells, which leads to rapid proliferation and tumor growth^38^,^39^. There are two main apoptosis pathways: the intrinsic pathway^40^ and the extrinsic pathway^41^. These pathways are mutually exclusive but are intricately linked to each other. The Bcl-2 family of proteins governs mitochondrial membrane permeability and can act in either a pro-apoptotic or anti-apoptotic manner via the intrinsic pathway^20^. Both the extrinsic and intrinsic pathway end at the point of the execution phase. Caspase-3 is considered to be the most important of the executioner caspases and is correlated with the activation of the apoptotic cascade^42^. After cisplatin treatment, variations in the levels of Bax and Bcl-2 proteins were observed. The reduced expression of Bcl-2 contributes to a loss of survival signals, but the increased expression of Bax can be an indicator of apoptosis via the intrinsic pathway. These results were similar to those found in uterine cervical cancer^43^, human gastric cancer MKN-45 cells, and human colon cancer LoVo cells^44^, in which apoptosis was induced by cisplatin with a reduction of Bcl-2 protein expression and an over-expression of Bax protein. It has also been reported that cisplatin concomitantly induces cell apoptosis with an increase in caspase-3 activity^45^, which was also established in our study. With the morphine (1 μg/ml) pretreatment, Bcl-2 expression increased over Bax, and a decrease in caspase-3 activity led to lower apoptosis in CNE-2 cells compared with the cisplatin treatment alone. This result suggests that the application of a low dose of morphine may be a potential reason for the increased resistance to cisplatin therapy observed in NPC patients. By contrast, a high dose of morphine (1000 μg/ml) improved the antitumor effects of cisplatin on CNE-2 cells by increasing the Bax/Bcl-2 ratio and the activity of caspase-3. A previous study reported that morphine treatment at concentrations of 1 μm and 10 μm resulted in chemoresistance to doxorubicin and paclitaxel by expanding the population of cancer stem cells in breast cancer^46^. In contrast, another study showed that 250 μm and 1250 μm doses of morphine can improve the antitumor effects of 5-Fluorouracil on MCF-7 breast cancer cells^47^. These results are similar to our results, which showed that the concentration of morphine maybe one reason for the different effects of morphine on chemotherapy. In addition, our results also demonstrated that other concentrations of morphine (0.1 μg/ml, 10 μg/ml and 100 μg/ml) did not affect cisplatin-induced CNE-2 cell apoptosis. These discrepancies are also associated with the different concentrations of morphine we used on the CNE-2 cell line. However, further studies should be performed to elucidate the mechanisms behind these effects.

Based on the results we obtained from our *in vitro* experiments and according to Gupta’s study^18^ and FDA guideline (http://www.fda.gov/ohrms/dockets/98fr/02d-0492-gdl0002.pdf), which shows HED (mg/kg) = Animal Dose (mg/kg) × [Animal Km/Human Km] (Human Km = 37,Mouse Km = 3), we tested the low dose of morphine (1 mg/kg) in an *in vivo* model. Based on this guideline, the dose used for mice in our experiment (1 mg/kg) is about 0.08 mg/kg for human beings. This single dose of morphine for iv is often used in clinical paitents, but it is very low dose for severe cancer pain patients. Meanwhile, although morphine has a short half-life of 1.5 - 7 hours, we administrated morphine every 2 days, not everyday or every several hours *in vitro* experiments. Because this model is more like clinical model. Actually, in clinical, it is uncommon for most early-stage cancer patients to receive morphine everyday or every several hours except for advanced cancer pain patients. We found that at this dose, morphine inhibited the cisplatin-induced apoptosis and reversed the cisplatin-inhibited tumor growth in CNE-2 xenograft-bearing mice. Because angiogenesis may participate in tumor progression^48^, we also examined the effects of morphine in a nasopharyngeal carcinoma model. We observed that morphine induced tumor neovascularization and reversed the cisplatin-induced decreases in neovascularization. However, morphine treatment did not increase tumor growth in mice treated with morphine alone. Other studies have shown similar results in a breast cancer model, in which morphine induced tumor neovascularization and also increased tumor volume on day 38^18^. We speculated that the discrepancies may have resulted from the different tumor characteristics and different durations of the experiments. Many clinical studies have established that the mu opioid receptor is independently associated with poor outcomes^49^,^50^. Nguyen J *et al.* showed increased mu opioid receptor expression in medium and large tumors but not in smaller tumors^51^. This may be another possible reason for the increased effect of morphine at a later period of tumor growth in our study. Recently, several retrospective clinical studies also raised the same concerns regarding the association of morphine requirements and poorer disease outcomes in lung^52^,^53^,^54^ and prostate cancer^50^.

Several xenograft mouse models have been generated to study the effects of morphine on cancer cell growth, with inhibiting tumor-growth results. In a melanoma mouse model generated via the subcutaneous injection of B16-BL6 cells into the hind paws of C57BL mice, morphine inhibited tumor growth and metastasis^55^. Harimaya Y *et al.* demonstrated that morphine inhibited tumor metastasis formation in a mouse model of colon cancer^56^. Another group showed that morphine inhibited tumor angiogenesis and tumor growth in a murine Lewis lung carcinoma cell tumor model^57^. These contrasting results are likely associated with the time of administration and/or the different concentrations of morphine. Only those that used chronic high doses of morphine demonstrated tumor suppression^55^,^56^,^57^, whereas the tumor-enhancing effects of morphine occurred after the administration of low daily doses or a single dose of morphine^18^,^19^,^58^.

In conclusion, our data provide the first evidence that the administration of a low dose of morphine causes chemoresistance to cisplatin in NPC both *in vitro* and *in vivo*. However, further clinical studies are needed to confirm our observations, and the potential mechanisms behind our observations are currently being pursued in our lab.

## Material and Methods

The experimental protocol was approved by the animal care committee of Sun Yat-sen University Cancer Center. The methods were carried out in accordance with the approved guidelines and regulations. The animals (athymic nude mice) used in this study were maintained in accordance with the Policy of Animal Care and Use Committee of Sun Yat-sen University Cancer Center.

### Cells, compound preparation and affinity

The human NPC cell line CNE-2 (a poorly differentiated NPC cell line) was originally obtained from NPC patients and maintained in our laboratory in RPMI 1640 medium (Gibco/Invitrogen, Gaithersburg, MD) supplemented with 10% heat-inactivated fetal bovine serum (HyClone/Thermo Fisher Scientific, Logan, UT). Cells were incubated in a humidified 5% CO_2_ atmosphere at 37 °C. Morphine hydrochloride was obtained from First Pharmacy Company of Shenyang.

For the *in vivo* experiments, CDDP and morphine were diluted in sterile normal saline solution for intraperitoneal administration. All dose formulations were prepared on the day of use.

### MTT assay

Cell viability was measured by a 3-[4,5-dimethylthiazol-2-thiazolyl]- 2,5-diphenyltetrazolium bromide (MTT) assay based on the mitochondrial conversion of MTT from a soluble tetrazolium salt into an insoluble colored formazan precipitate, which was dissolved in DMSO and quantitated by spectrophotometry (Thermo Multiskan MK3; Thermo Labsystems, Vantaa, Finland) to obtain optical density (OD) values. Serial dilutions were made from a stock solution of morphine or CDDP to the desired concentrations. Briefly, CNE2 cells were plated in 96-well culture clusters (Costar, Cambridge, MA) at a density of 25,000 cells/mL in triplicate. Morphine (0.1 μg/ml, 1 μg/ml, 10 μg/ml, 100 μg/ml, 1000 μg/ml) or cisplatin (4 μg/ml) was added at 24, 48, or 72 h. Morphine was added 1 h before the administration of CDDP and 20 μL of 5 mg/mL MTT was added 4 h prior to the time points when 150 μL of DMSO was added to each well. The absorbance values are represented as a percentage of the unknown samples relative to the control samples and plotted as a linear function of drug concentration. The 50% inhibitory concentration (IC_50_) was identified as the concentration of drug required to achieve a 50% growth inhibition relative to the control populations. The inhibition of cell growth was measured as a percentage of viable cells relative to the control and was calculated as follows: % viable cells rate = 100% × OD_T_/OD_C_, where OD_T_ is the average OD value of the treated samples, and OD_C_ is the average OD value of the control samples.

### Annexin V-FITC/PI apoptosis detection by flow cytometry

CNE2 cells were treated with cisplatin (4 μg/ml), cisplatin (4 μg/ml) and morphine (0.1 μg/ml, 1 μg/ml, 10 μg/ml, 100 μg/ml, 1000 μg/ml), or saline for 72 h. Morphine was added 1 h before CDDP administration. Cisplatin and/or morphine induced apoptosis in tumor cells was evaluated using an Annexin V–FITC apoptosis assay. The cells were cultured in a six-well plate and were exposed to cisplatin and/or morphine for the indicated times. For the Annexin V–FITC apoptosis assay, the cells were collected and resuspended with 10% RPMI 1640 medium at the density of approximately 1 × 10^6^ cells/mL. Then, the cells were incubated at room temperature in the presence of media binding reagent and Annexin V–FITC for 15 min in the dark. After being washed in PBS, the cells were resuspended in cold 1 × binding buffer and 10 μL propidium iodide (30 μg/mL) were added. The samples were placed on ice and away from light. Stained cells were immediately subjected to analysis by flow cytometry (Beckman Coulter, Cytomics FC 500, CA) at the wavelength of 488 nm.

### Western blot analysis

CNE2 cells were seeded in six-well plates at 2 × 10^5^ cells/well and were incubated overnight. They were then treated with 4 μg/ml cisplatin for 72 h, with 4 μg/ml cisplatin in the presence of 1 μg/ml morphine or 1000 μg/ml morphine for 72 h, or with vehicle (used as a control). The cells were lysed using a solution containing 50 nM Tris (pH 7.5), 150 mM NaCl, and 0.5% NP-40 on ice. Fifty micrograms of total protein from each sample were resolved on a 12% Bis–Tris gel with MOPs running buffer and were transferred to nitrocellulose membranes. The blots were probed with various antibodies, including anti-β-actin (1: 3000, Abcam, ab179467), anti-Bax (1:500, Abcam, ab7977), anti-Bcl-2 (1:500, Abcam, ab32124), anti-caspase-3 (1:300, Abcam, ab44976) and anti-cleaved-caspase-3 (1:200, Cell Signaling Technology, 9661 ).

### *In vivo* experiments, immunohistochemistry and TUNEL staining assay

Five-week-old athymic nude (nu/nu) mice were obtained from the Animal Center of Southern Medical University (Guangdong, China) and were housed in isolation in pathogen-free, ventilated cages in a temperature-controlled room (22–25 °C) with a 12–12 h light/dark cycle (light on 07:00–19:00). The mice received subcutaneous injections of CNE2 cells (1 × 10^7^) in the axillary area. The mice were euthanized when the subcutaneous tumors were approximately 1500 mg. The tumors were dissected and mechanically dissociated into equal pieces that could then be transplanted into the flanks of a new group of mice. The mice were checked every 2 days for xenograft development. When the tumors became palpable (approximately 0.1 mm^3^), the mice were randomly divided into several groups. Each group contained 10 mice, and there was no difference in tumor size between the groups. Using this model, we monitored the efficacy of cisplatin alone (3 mg/kg of body weight, given by intraperitoneal injection every 2 days), saline alone (0.9% NS, 0.1 ml/10 g, given by intraperitoneal injection every 2 days), morphine alone (1 mg/kg body weight, given by intraperitoneal injection every 2 days) or combined cisplatin and morphine. The tumor volumes were recorded using the following formula: (a × b^2^)/2, where a and b represent the tumor length and width (in mm), respectively. The body weights were also recorded.

Immunohistochemical analysis and terminal deoxynucleotidyl transferase biotin-dUTP nick-end labeling (TUNEL) staining were performed on tissue sample sections of CNE-2 xenografts obtained from 4 treatment groups: saline solution (control), morphine, CDDP, and the combined treatment of morphine and CDDP. All samples were stained with hematoxylin and eosin and were microscopically examined to confirm an NPC cell origin. The sections were deparaffinized, rehydrated using xylene and alcohol, and stained with cleaved-caspase-3 antidody (Cell Signaling ) and CD34 antibody (Abcam) at 4 °C overnight or for TUNEL at 37 °C for 60 min. All antibodies and TUNEL labels were visualized using diaminobenzidine (DAB) (DAKO Liquid DAB, Dako, Carpinteria, CA) as a peroxidase substrate. TUNEL-positive nuclei were stained brown (DAB), and all other nuclei were stained blue^59^.

Two skilled pathologists independently reviewed the slides and recorded the proportion of stained cells in each treatment group. The measurements of apoptosis cells by counting the numbers of TUNEL-positive cells or the cleaved-caspase-3-positive cells per × 40 high-power field (HPF). Five different fields were randomly chose per section. And the measurements of MVD by counting the CD34-stained vessels under light microscopy. Regions with the highest vessel density (“hot spots”) were located by scanning the tissue sections under × 4-power microscope. After identification of the “hot spots”, five different fields were randomly chosen within each hot spot per section, and each endothelial cell or cell cluster that showed antibody staining and that was clearly separated from adjacent clusters was counted at per × 40 high-power field (HPF) for MVD measurements. The average numbers of the five different fields were recorded. The average of the two observers’ results was used for statistical analysis.

### Statistical analysis

The data are presented as the mean values ± SD and were analyzed using SPSS 22.0 software (SPSS, Inc, Chicago, IL). Differences between single group mean values with control mean values were analyzed using a one tailed Student’s *t*-test and either one-way or two-way analysis of variance (ANOVA) where appropriate. Differences were considered statistically significant at p < 0.05.

## Additional Information

**How to cite this article**: Cao, L.-H. *et al.* Morphine, a potential antagonist of cisplatin cytotoxicity, inhibits cisplatin -induced apoptosis and suppression of tumor growth in nasopharyngeal carcinoma xenografts. *Sci. Rep.* **6**, 18706; doi: 10.1038/srep18706 (2016).
